# Interpreting ambiguous ‘trace’ results in *Schistosoma mansoni* CCA Tests: Estimating sensitivity and specificity of ambiguous results with no gold standard

**DOI:** 10.1371/journal.pntd.0006102

**Published:** 2017-12-08

**Authors:** Michelle N. Clements, Christl A. Donnelly, Alan Fenwick, Narcis B. Kabatereine, Sarah C. L. Knowles, Aboulaye Meité, Eliézer K. N'Goran, Yolisa Nalule, Sarah Nogaro, Anna E. Phillips, Edridah Muheki Tukahebwa, Fiona M. Fleming

**Affiliations:** 1 Schistosomiasis Control Initiative, Imperial College, London, United Kingdom; 2 MRC Centre for Outbreak Analysis and Modelling, Department of Infectious Disease Epidemiology, Imperial College London, London, United Kingdom; 3 Schistosomiasis Control Initiative, Imperial College, Kampala, Uganda; 4 The Royal Veterinary College, Hawkshead Lane, Hatfield, Hertfordshire, United Kingdom; 5 Côte d'Ivoire Ministry of Health, National Program Against Filariasis, Schistosomiasis and Geohelminths, Abidjan, Côte d'Ivoire; 6 Unité de Formation et de Recherche Biosciences, Université Félix Houphouët-Boigny, Abijan, Côte d'Ivoire; 7 Vector Control Division, Ministry of Health Uganda, Kampala, Uganda; Swiss Tropical and Public Health Institute, SWITZERLAND

## Abstract

**Background:**

The development of new diagnostics is an important tool in the fight against disease. Latent Class Analysis (LCA) is used to estimate the sensitivity and specificity of tests in the absence of a gold standard. The main field diagnostic for *Schistosoma mansoni* infection, Kato-Katz (KK), is not very sensitive at low infection intensities. A point-of-care circulating cathodic antigen (CCA) test has been shown to be more sensitive than KK. However, CCA can return an ambiguous ‘trace’ result between ‘positive’ and ‘negative’, and much debate has focused on interpretation of traces results.

**Methodology/Principle findings:**

We show how LCA can be extended to include ambiguous trace results and analyse *S*. *mansoni* studies from both Côte d’Ivoire (CdI) and Uganda. We compare the diagnostic performance of KK and CCA and the observed results by each test to the estimated infection prevalence in the population.

Prevalence by KK was higher in CdI (13.4%) than in Uganda (6.1%), but prevalence by CCA was similar between countries, both when trace was assumed to be negative (CCAtn: 11.7% in CdI and 9.7% in Uganda) and positive (CCAtp: 20.1% in CdI and 22.5% in Uganda). The estimated sensitivity of CCA was more consistent between countries than the estimated sensitivity of KK, and estimated infection prevalence did not significantly differ between CdI (20.5%) and Uganda (19.1%). The prevalence by CCA with trace as positive did not differ significantly from estimates of infection prevalence in either country, whereas both KK and CCA with trace as negative significantly underestimated infection prevalence in both countries.

**Conclusions:**

Incorporation of ambiguous results into an LCA enables the effect of different treatment thresholds to be directly assessed and is applicable in many fields. Our results showed that CCA with trace as positive most accurately estimated infection prevalence.

## Author summary

Schistosomiasis is a debilitating disease affecting over 200 million people worldwide, mainly in developing countries. Treatment for schistosomiasis is straightforward and involves treating all school-age children, and sometimes also adults, in areas where schistosomiasis is known to occur. However, detecting intestinal schistosomiasis (*Schistosoma mansoni)* using Kato-Katz, the most common technique, can fail to detect infection when it is present as Kato-Katz relies on finding eggs within a small stool sample. A new field diagnostic, CCA, is promising as it is simple to use and seems to detect more infections that Kato-Katz. However, assessing the performance of CCA is difficult as we cannot be certain whether or not those positive by CCA but negative by Kato-Katz are truly infected. Additionally, CCA can often return a trace result between negative and positive which is difficult to interpret. We assess the performance of Kato-Katz and CCA in both Côte d’Ivoire and Uganda. We showed that CCA did indeed detect more infections than Kato-Katz, and that CCA accurately estimated the proportion of people truly infected in the population, when trace readings were considered positive. CCA is consequently an important and valuable tool in the fight against schistosomiasis.

## Introduction

It is estimated that 237 million individuals require treatment for schistosomiasis [1]. Endemic in 56 countries spanning over Africa, the Middle East, South America, and the West Indies, *Schistosoma mansoni* is the most geographically widespread schistosome species. Nevertheless, despite a growing body of studies looking at distribution (e.g. [2–4]), we do not have a true representation of the number of people infected with *S*. *mansoni* as there is no definitive ‘gold standard’ field diagnostic test. Accurate prevalence estimates of those infected are important, as even low infection intensities have associated morbidity [5]. The current recommended control method for schistosomiasis is preventive chemotherapy (PC) of at-risk populations, where all school-aged children (SAC) and, where appropriate, at-risk adults in the community are treated. Frequency of treatment and who receives the drugs are dependent on the prevalence of schistosomiasis in the local area [6], as determined by the parasitological diagnostic test Kato-Katz, where eggs are detected in faecal samples examined microscopically [7, 8]. Kato-Katz has low sensitivity in those with low infection intensities and in areas of low prevalence, as egg output varies both within and between days [9] and infection can easily be missed [10]. However, Kato-Katz is highly specific, as an *S*. *mansoni* egg is easily identifiable to a trained technician [11].

A rapid diagnostic test, Circulating Cathodic Antigen (CCA), has recently been endorsed by the World Health Organisation (WHO) for use in mapping and programme impact evaluation [12]. CCA uses a urine sample to test for *S*. *mansoni* infection and consequently is much simpler to use than Kato-Katz. However, the presence of an ambiguous result between negative and positive, known as a ‘trace’ result, complicates the interpretation of a CCA test. There is no consensus in the literature on whether trace should be considered as positive or negative (e.g. trace as negative as found to be closest to Kato-Katz:[13], trace assumed to be negative: [14], trace assumed to be positive: [11], [15]). Additionally, it is not clear how to interpret prevalence estimates from CCA tests and whether or not they are reflective of infection prevalence in the population.

Latent Class Analysis (LCA) estimates the sensitivity and specificity of diagnostic tests and the prevalence in the population in the absence of a gold standard, and has been applied in a wide range of fields including human soil transmitted helminthiases [16], malaria [17], and veterinary biology [18]. Analysis of LCA can be within a frequentist or Bayesian framework, with the Bayesian approach having several advantages. Firstly, the distribution of additional parameters such as Positive and Negative Predictive Values (PPV and NPV respectively) are easily calculated from the posterior distributions obtained from the LCA, which enables the results to be more easily interpreted. Secondly, Bayesian analysis enables straightforward comparison of estimated infection prevalence and the prevalence by each test to assess how well the test performs in estimating prevalence rather than infection status of each individual. Finally, the use of posterior distributions enables straightforward testing of differences between countries and between tests.

The aim of this study is to robustly analyse CCA data from two countries, Côte d’Ivoire and Uganda, to determine the effects of considering CCA trace as negative or positive. We particularly focus on assessing the performance of CCA and Kato-Katz at measuring ‘infection prevalence’, which is estimated from the LCA, and is the main use of *S*. *mansoni* diagnostics in control programs.

## Methods

### Ethics statement

Ethical approval for both surveys, including the consent process, was obtained from Imperial College Research Ethics Committee (ICREC_8_2_2) as well as from the appropriate country: Comité National d’Ethique de la Recherche (CNER; ref: 086/MSHP/CNER-kp) in Côte d’Ivoire and Uganda National Council for Science and Technology (UNCST; ref: HS1993) in Uganda.

The surveys were undertaken as part of the national schistosomiasis control programmes in each country, overseen and approved by the relevant Ministries of Health. As participants were under 18 years of age, written consent was required by a parent or informed guardian. Head-teachers in each school acted as the informed guardian as literacy levels in many areas are low. The head-teacher was informed fully about the study and requested to provide informed consent for field teams to collect urine and stool samples from pupils. Only children who consented orally both before and after selection in the presence of a witness (head-teacher) took part in the survey. Additionally, all children gave urine and stool samples freely following selection, and there were no consequences if a child did not return their samples. All data were entered and analysed anonymously.

### Data collection

#### Overall summary

Data were collected as part of the programme Monitoring and Evaluation activities within each country. Samples were collected as part of a Monitoring & Evaluation impact study, in which a consistent age range of children in a set of schools (sentinel sites) are screened for schistosomiasis over multiple years to estimate changes in prevalence and intensity of infection over time. In Côte d’Ivoire data collected in 2013 were ‘baseline’ data, that is, data collected in the first year of the control programme prior to mass treatment with Praziquantel. In contrast, Uganda established a national schistosomiasis control programme in 2003 and had received at least four rounds of PC prior to the survey in 2013. In addition, schools selected in Uganda were part of an operational research project where three different treatment strategies in years following the baseline survey were randomly allocated to each of the schools. Data were collected between 28^th^ October– 12^th^ November 2013 in Côte d’Ivoire and between 30^th^ September– 16^th^ October 2013 in Uganda. Full study protocols are available on request from the Schistosomiasis Control Initiative, Imperial College London.

#### Selection of schools

Data were collected in 26 schools across 10 districts in Côte d’Ivoire and in 14 schools across four districts in Uganda. The surveyed schools were randomly selected from the sampling frame in each country.

In Côte d’Ivoire, all schools in the baseline survey were tested with both CCA and Kato-Katz, and the sample size calculation was powered to be able to detect change in prevalence between the baseline and repeat surveys using Kato-Katz and not for the purposes of diagnostic comparison. In Côte d’Ivoire the sampling frame was all schools involved in a baseline schistosomiasis mapping survey in 2012 where at least one child infected with *S*. *mansoni* was observed during mapping, within districts that fell into moderate (≥ 10% and <50% prevalence by Kato-Katz) or high (≥ 50% prevalence by Kato-Katz) prevalence categories by WHO guidelines [19]. Mapped schools were used in the site selection as no treatment had occurred in these schools post-mapping.

In Uganda, 120 schools were selected to be were sampled using CCA only, with 40 schools being in each of three treatments arms, although in the field only 119 schools were sampled. A subset of 14 schools (planned to be 15) were sampled using both CCA and Kato-Katz to obtain a pre-determined sample size of approximately 300 pupils in each treatment arm to enable comparison of test results using LCA; here we focus on the 14 schools only. The sampling frame for Uganda was a list of all mixed-sex primary schools with at least 100 pupils in ‘low endemic’ sub districts from the 2012 CCA mapping exercise in Uganda, that had received at least four rounds of praziquantel treatment. Schools that were mapped as part of the recent mapping exercise where no schistosomiasis was found with CCA were excluded from the sampling frame and ‘low endemic’ was defined as those subcounties with equivalent to less than 10% *S*. *mansoni* prevalence by Kato-Katz, back calculated as 1–46% by CCA [20].

#### Selection of children within schools

In Côte d’Ivoire, 125 children per school from classes CP1, CP2 and CE1 (roughly equivalent to 6, 7 and 8-year-olds) were targeted for sampling. In Uganda, 60 students were targeted in each school although sampling of children by grades was not consistent. Nine schools sampled children in primary classes 1, 4, 5 and 6 (roughly equivalent to 6, 9, 10 and 11-year-olds) as per the sampling protocol and five schools deviated from the sampling protocol by sampling children in grades 1, 3, 5 and 7 (roughly equivalent to 6, 8, 10 and 12-year-olds). In both countries, equal number of boys and girls were randomly sampled within each school, selection of pupils within each class and sex was random, and there was no reference to possible infection status during selection. There was no treatment of the children during the surveys as treatment was part of the wider school-based PC treatment administered by the national programs following the surveys whereby all children were treated regardless of infection status.

#### Parasitological variables

Duplicate Kato-Katz thick smear slides per stool sample (using 41.7 mg template) were prepared and examined for *S*. *mansoni* using WHO approved standard operating procedures on each of two and three consecutive days in Côte d’Ivoire and Uganda, respectively. To provide a like-for-like comparison, we included data collected on only the first two days from Uganda in results presented here. A child was defined to be positive by Kato-Katz if one or more *S*. *mansoni* eggs were found on any of the four slides examined, and mean infection intensity in eggs per gram (epg) was calculated by multiplying the average number of eggs found across all examined slides by 24.

CCA data were collected on the first day of the survey in Côte d’Ivoire and on all three days in Uganda; for comparison purposes only CCA results from the first day of data collection have been assessed in both countries. CCA results were scored on a four-point scale in Côte d’Ivoire and a five-point scale in Uganda, with 0 denoting negative and the lowest positive score denoting trace, as per manufacturers recommendation, in both countries. A child was defined to be positive by CCA with trace assumed negative (CCAtn) if the CCA result was greater than trace in either country, and positive by CCA with trace assumed positive (CCAtp) if the CCA result was greater than or equal to trace in either country. Only children with four slides of Kato-Katz (two slides on each of two days) and one CCA result were included in the analyses.

Quality control was assured through rigorous training before the field survey and quality checks during the field survey. In Côte d’Ivoire, each Kato-Katz slide was read independently by two slide technicians, and any differences greater than 10% were reread by a third person to determine the correct egg count. Additionally, 10% of the slides were checked for quality control, and the CCA readings were always read by the same person to ensure consistency across time. In Uganda, an experienced technician rescored at least 10% of all Kato-Katz slides, and independently performed CCA tests on at least 10% of all urine samples. Kato-Katz and CCA test processing was performed by the same survey team members in each school. The tests were numbered and the Kato-Katz and CCA tests were processed separately. Consequently, clinical information and other tests results were not available to the field team during test processing. Testing required only urine and stool samples from each child and no adverse effects from performing either test were observed.

### Statistical analysis

#### Latent class analysis

Analysis of the test results was by Bayesian LCA. Full details of the methodology are available in the S1 Supporting Information and consequently we focus on the main points here. All analyses were pre-specified. LCA estimates the sensitivity and specificity of each test and the prevalence in the population. Sensitivity is defined as the proportion of people truly positive testing positive and specificity is defined as the proportion of people truly negative testing negative, with high values of both parameters being desirable.

The LCA firstly involves calculating the number of people with each possible test combination, which is then related to the sensitivity and specificity of each test and the prevalence, using likelihood functions. For example, the number of people testing positive on two tests can be split into two groups–those who are truly positive (with prevalence being the proportion of individuals in this group) and correctly test positive on both tests (related to the sensitivities of the tests), and those who are truly negative and incorrectly test positive on both tests (related to the specificities of the tests); see S1 Supporting Information for full explanation and equations). Similar logic can be used for each possible test combination and these equations are then tied to the observed numbers using a multinomial distribution.

The simplest form of LCA assumes that tests are independent conditional on true infection status. That is, within each group of positive or negative people, knowing the results of one test does not allow you to predict the results of any other test. This can be violated if, for example, people who are heavily infected are more likely to test positive on both tests than those who are lightly infected. Dendukuri and Joseph, 2001 [21], proposed an extension to the basic LCA where covariance terms are added and, in a Bayesian framework, limits are placed on the prior distributions so that proportions are always between zero and one.

Incorporating trace results into the model requires adding an additional result to the positive or negative normally assumed for each test. However, there is also an important insight required to construct the likelihood functions. Taking CCA trace as positive (CCAtp) will lead to more people being positive than when trace is negative (CCAtn). If at least one of these trace results is truly positive then the sensitivity of CCAtp will be more than the sensitivity of CCAtn, and if at least one of these trace results is truly negative then the specificity of CCAtp will be less than the specificity of CCAtn. We can combine the above to give the following likelihood equations:

$$p_{++}=prev*{(Se}_{1}*{Se}_{2}+{covSe}_{1,2})+(1-prev)*((1-{Sp}_{1})*(1-{Sp}_{2})+{covSp}_{1,2})$$ (1)

$$p_{+t}=prev*{(Se}_{1}*adj{Se}_{2}{+covSe}_{1,adj})+(1-prev)*((1-{Sp}_{1})*adj{Sp}_{2}{+covSp}_{1,adj})$$ (2)

$$p_{+-}=prev*{(Se}_{1}*{(1-(Se}_{2}+adj{Se}_{2}))-({covSe}_{1,2}+{covSe}_{1,adj}))+(1-prev)*((1-{Sp}_{1})*{(Sp}_{2}-adj{Sp}_{2})-({covSp}_{1,2}-{covSp}_{1,adj}))$$ (3)

$$p_{-+}=prev*{((1-Se}_{1})*{Se}_{2}-{covSe}_{1,2})+(1-prev)*{(Sp}_{1}*(1-{Sp}_{2})-{covSp}_{1,2})$$ (4)

$$p_{-t}=prev*{((1-Se}_{1})*adj{Se}_{2}-{covSe}_{1,adj})+(1-prev)*{(Sp}_{1}*adj{Sp}_{2}{-covSp}_{1,adj})$$ (5)

$$p_{--}=prev*((1-{Se}_{1})*{(1-(Se}_{2}+adj{Se}_{2}))+({covSe}_{1,2}+{covSe}_{1,adj}))+(1-prev)*{(Sp}_{1}*{(Sp}_{2}-adj{Sp}_{2})+({covSp}_{1,2}-{covSp}_{1,adj}))$$ (6)

where *p*_*KC*_ denotes the proportion of people with a given test combination for Kato-Katz (*K*) and CCA (*C*)–with Kato-Katz results being positive (+) or negative (-) and CCA results being positive (+), trace (t) or negative (-); *Se*_1_ *Sp*_1_, *Se*_2_ and *Sp*_2_ denote the sensitivity and specificity of Kato-Katz and CCAtn respectively; *adjSe*_2_ and *adjSp*_2_ denotes how much more sensitive and less specific, respectively, CCA becomes when trace is considered as positive rather than negative; and *prev* denotes the infection prevalence in the population. The sensitivity of CCAtp is then equal to:

$${Se}_{2}+adj{Se}_{2}$$ (7)

and the specificity of CCAtp equals:

$${Sp}_{2}-adj{Sp}_{2}$$ (8)

In a similar manner to Dendukuri and Joseph (2001), we place limits on the covariances:

$${({Se}_{1}-1)(1-{Se}_{2})\leq covSe}_{1,2}\leq \min\left({Se}_{1},{Se}_{2}\right)-{Se}_{1}*{Se}_{2}$$ (9)

$${({Se}_{1}-1)(1-{Se}_{adj})\leq covSe}_{1,adj}\leq \min\left({Se}_{1},{adjSe}_{2}\right)-{Se}_{1}*{adjSe}_{2}$$ (10)

$${{-Se}_{1}*({{Se}_{2}+adjSe}_{2})\leq {covSe}_{1,2}+covSe}_{1,adj}\leq \min\left({1-Se}_{1},{1-({Se}_{2}+adjSe}_{2})\right)-{(1-Se}_{1})*({1-({Se}_{2}+adjSe}_{2})$$ (11)

$${({Sp}_{1}-1)(1-{Sp}_{2})\leq covSp}_{1,2}\leq \min\left({Sp}_{1},{Sp}_{2}\right)-{Sp}_{1}*{Sp}_{2}$$ (12)

$${({Sp}_{1}-1)(1-adj{Sp}_{2})\leq covSp}_{1,adj}\leq \min\left({Sp}_{1},{adjSp}_{2}\right)-{Sp}_{1}*adj{Sp}_{2}$$ (13)

$${({Sp}_{1}-1)*({1-({Sp}_{2}-adjSp}_{2}))\leq {covSp}_{1,2}-covSp}_{1,adj}\leq \min\left({Sp}_{1},{{Sp}_{2}-adjSp}_{2}\right)-{Sp}_{1}*{{(Sp}_{2}-adjSp}_{2})$$ (14)

and we also place limits on *adjSe*_2_ and *adjSp*_2_ so that the sensitivity and specificity of *Se*_2_ + *adjSe*_2_ and *Sp*_2_ − *adjSp*_2_ remain between 0 and 1:

$$0{\leq adjSe}_{2}\leq 1-{Se}_{2}$$ (15)

$$0{\leq adjSp}_{2}\leq {Sp}_{2}$$ (16)

The data are then linked to Eqs 1–6 through a multinomial distribution where *N* is the total number of people tested:

$$\left(n_{++,}n_{+t},n_{+-},n_{-+},n_{-t},n_{--})∼Multinomial(p_{++,}p_{+t},p_{+-},p_{-+},p_{-t},p_{--},N\right)$$ (17)

#### Running the LCA

The LCA described above was run in OpenBugs [22] using the R2OpenBugs package [23] in R version 3.2.2 [24]. Analysis was performed separately for each country, taking each school to be a separate population [16]. Beta prior distributions were used for sensitivities and specificities. We imposed the strongest prior distribution on the specificity of Kato-Katz (Beta(α = 21.2, β = 2.06), which is equivalent to 95% certain greater than 80%, mode at 95%), as the presence of an egg confirms infection, and weaker prior distributions on all other sensitivities and specificities. For the sensitivity of both Kato-Katz and CCAtn, we used a prior of Beta(3.05, 1.15) which is equivalent to 95% certain bigger than 30% with a mode at 80%, and for the specificity of CCAtn we used a prior of Beta(5.38, 1.49) which is equivalent to 95% certain bigger than 50% with a mode at 90%. For covariances and adjusted values of CCA trace positive sensitivity and specificity relative to CCA trace negative, we used uniform prior distributions on the restricted values described above, to ensure proportions remained bounded between zero and one, with the exception that all covariances were restricted to be positive. We incorporated the limits on the sum of the covariances into the model by adding extra restrictions to the individual covariances so that their sum was limited to the required amount. We ran three MCMC chains, where the starting points were randomly sampled, for 3,000 iterations following a burn-in of 1,000 with a thin of 25, and checked for convergence between the chains using Gelman diagnostics [25]. Following the recommendation of Collins and Huynh 2014 [26], we imposed the restriction of sensitivity plus specificity of each test being greater than 1 to avoid issues associated with label switching. The code for running the LCA is available in S2 Supporting Information.

Sensitivity and specificity of CCA with trace as positive were calculated for each of the 6,000 stored iterations so that the distribution of these metrics could be obtained. We compared the sensitivities and specificities, both from different countries and different tests, by calculating the distribution of the difference between the focal metrics for each of the 6,000 iterations and determining if the estimate was significantly different from zero using the 95% Bayesian credible interval (BCI).

#### Infection prevalence, PPV and NPV

An estimate of infection prevalence was obtained by averaging the estimated infection prevalence in each school, weighted for the number of pupils within each school. This overall infection prevalence estimate was used to obtain distributions for the probability of an individual testing positive being truly positive (positive predicted value; PPV) and the probability of an individual testing negative being truly negative (negative predicted value; NPV) using the standard equations below:

$${PPV}_{i}=\frac{prev*{Se}_{i}}{prev*{Se}_{i}+(1-prev)*(1-{Sp}_{i})}$$ (18)

$${NPV}_{i}=\frac{(1-prev)*{Sp}_{i}}{prev*{(1-Se}_{i})+(1-prev)*{Sp}_{i}}$$ (19)

where *i* denotes each separate test, and *prev* denotes the overall prevalence estimated above.

#### Comparison between infection and test prevalence

We obtained a distribution for test prevalence for each test (KK, CCAtn and CCAtp) in two steps. Firstly, we obtained a distribution for the number of children positive within each school for each test by sampling 6,000 times (the number of iterations in the posterior distribution) from a Binomial distribution with parameters *n* equal to the number of children tested within the school and *p* equal to the observed prevalence by the focal test in the school. Secondly, we calculated overall test prevalence for each of the 6,000 iterations by summing the total number of children positive in each school by the focal test and dividing by the total number of children tested. The difference between infection and test prevalence was then calculated through simple subtraction on each of the 6,000 iterations to obtain a distribution of the specified differences.

## Results

### Descriptive results

A total of 3,035 and 693 children were included in the analysis for Côte d’Ivoire and Uganda, respectively (Table 1). Prevalence by Kato-Katz was higher in Côte d’Ivoire (13.4%) than in Uganda (6.1%), with mean intensity of infection, among all children, being over seven-fold higher in Côte d’Ivoire (26.8 eggs per gram (epg)) than in Uganda (3.4 epg; Table 1). However, prevalence by CCA was similar in both countries, both when trace was assumed to be negative (CCAtn prevalence 11.7% in Côte d’Ivoire and 9.7% in Uganda) and when trace was assumed to be positive (CCAtp prevalence 20.1% in Côte d’Ivoire and 22.5% in Uganda; Table 1). A completed STARD checklist and participant flow diagram are available in S3 and S4 Supporting Informations respectively and the raw data used for analyses are available in S5 (Côte d’Ivoire) and S6 (Uganda) Supporting Informations.

**Table 1:** Summary statistics for Côte d’Ivoire and Uganda. KK denotes Kato-Katz.

		Côte d’Ivoire	Uganda
Number schools		26	14
Number pupils		3035	693
Number age missing		116	22
Mean age in years (±SD)		7.9 (±1.6)	9.9 (±2.6)
Age range in years		4–15	5–17
Number sex missing		0	0
Number female		1485	268
Proportion female		49%	39%
Prevalence by Kato-Katz		13.4%	6.1%
Prevalence by CCAtn		11.7%	9.7%
Prevalence by CCAtp		20.1%	22.5%
Mean epg by Kato-Katz (±SD)		26.9 (±167.1)	3.4 (±29.0)
			
*Proportion of test combinations*			
KK Result	CCA result	Côte d’Ivoire	Uganda
positive	positive	6.5%	3.8%
positive	trace	2.2%	0.9%
positive	negative	4.7%	1.4%
negative	positive	5.2%	5.9%
negative	trace	6.2%	12.0%
negative	negative	75.2%	76.0%

**Fig 1:**
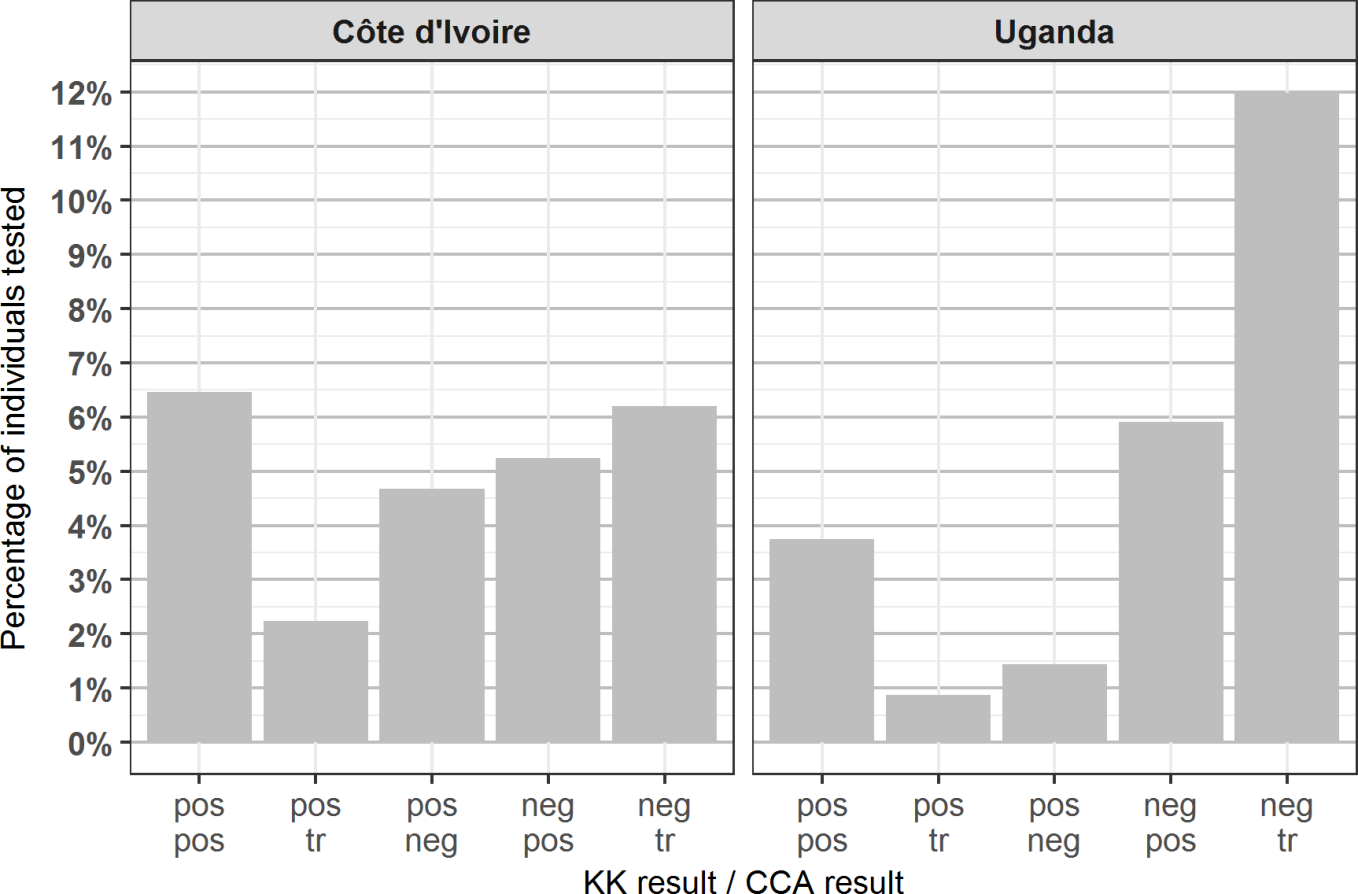
Bar chart showing the proportion of children tested in each country with specified test results. For example, ‘pos/pos’ denotes the proportion of children in each country testing positive for both tests, and ‘pos/tr’ denotes the proportion of children in each country testing positive by Kato-Katz and trace by CCA. Note that the bar showing the proportion of children negative on both tests (75% in Côte d’Ivoire and 76% in Uganda) has been removed from the graph to enable easier comparison of non-negative results.

In both countries, just over 75% of pupils were negative on both tests, and 6.5% of children in Côte d’Ivoire and 3.8% of children in Uganda were positive on both tests (Table 1, Fig 1). CCA sometimes failed to detect infection where eggs were found by Kato-Katz: 4.7% of pupils in Côte d’Ivoire and 1.4% of pupils in Uganda had a negative CCA result but were positive by Kato-Katz. Similarly, Kato-Katz sometimes failed to detect infections that were positive (not trace) by CCA: 5.2% and 5.9% of pupils in Côte d’Ivoire and Uganda, respectively, tested positive for CCA but negative by Kato-Katz. Tables of CCA results split by Kato-Katz infection category are available in S7 Supporting Information.

### Latent class analysis

**Fig 2:**
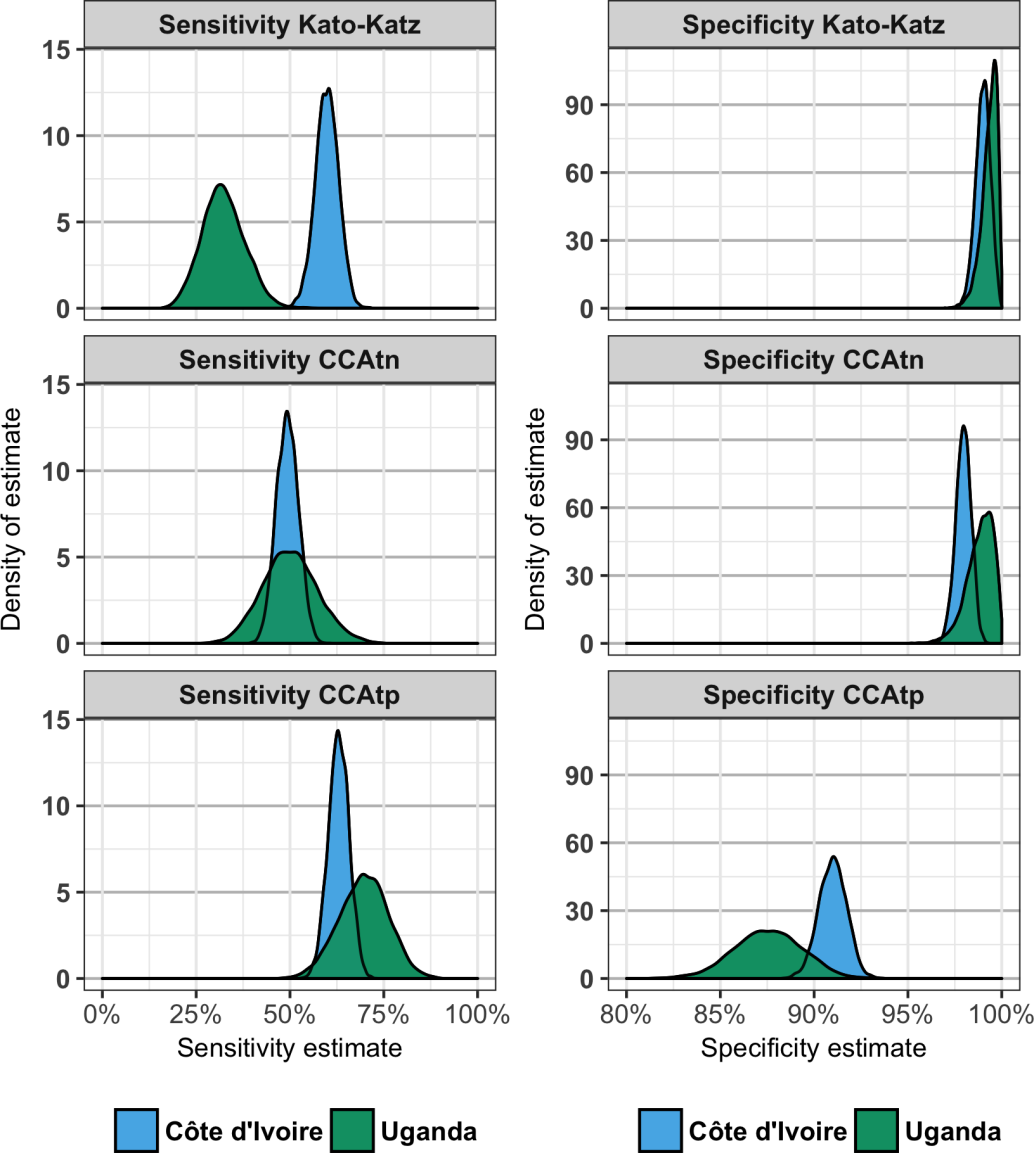
Plots showing the posterior distributions of sensitivity (left) and specificity (right) of each test in Côte d’Ivoire (blue) and Uganda (green) estimated from a Bayesian Latent Class Analysis. Note that the x-axis for the specificity has been truncated to begin at 80% to enable easier comparison between tests.

Estimates of parameters obtained from the Bayesian LCA (sensitivity and specificity of each test and prevalence) with the associated BCI are available in Table 2, and Fig 2 shows the posterior distributions of sensitivity and specificity of each test. Table 2 also shows parameters calculated from the posterior distributions of the estimated parameters.

**Table 2:** Output from Bayesian LCA showing mean estimate and the 95% Bayesian credible interval. Sensitivity and specificity and infection prevalence were estimated from the model while all other parameters were estimated from these posterior distributions using the equations described in the methods. The column on the right shows the estimate of the difference between the Côte d’Ivoire and Uganda estimates.

	Côte d’Ivoire	Uganda	Côte d’Ivoire - Uganda
**Sensitivity**	** **	** **	** **
Kato-Katz	59.9% (53.6%, 65.7%)	32.3% (21.8%, 43.9%)	27.5% (14.2%, 39.8%)
CCAtn	49.3% (43.6%, 55.0%)	50.1% (36.2%, 65.1%)	-0.8% (-17.0%, 14.1%)
CCAtp	63.0% (57.5%, 68.3%)	69.7% (56.4%, 82.0%)	-6.7% (-20.3%, 7.4%)
CCAtn - Kato-Katz	-10.5% (-17.0%, -4.1%)	17.8% (5.6%, 30.9%)	
CCAtp - Kato-Katz	3.1% (-3.4%, 9.5%)	37.3% (24.5%, 49.7%)	
CCAtp - CCAtn	13.7% (10.3%, 17.4%)	19.5% (10.2%, 30.0%)	
**Specificity**	** **	** **	
Kato-Katz	99.0% (98.2%, 99.7%)	99.3% (98.3%, 99.9%)	-0.3% (-1.4%, 0.9%)
CCAtn	98.0% (97.1%, 98.7%)	98.8% (97.2%, 99.9%)	-0.9% (-2.3%, 0.9%)
CCAtp	87.6% (83.9%, 91.1%)	91.0% (89.5%, 92.4%)	3.4% (-0.4%, 7.4%)
CCAtn - Kato-Katz	-1.0% (-2.1%, 0.0%)	-0.5% (-2.2%, 0.8%)	
CCAtp - Kato-Katz	-8.0% (-9.7%, -6.4%)	-11.8% (-15.5%, -8.2%)	
CCAtp - CCAtn	-7.0% (-8.2%, -5.8%)	-11.3% (-14.6%, -8.1%)	
**Infection prevalence**	** **	** **	
Infection prevalence	20.5% (18.4%, 22.9%)	19.4% (14.8%, 25.0%)	1.2% (-4.9%, 6.4%)
**PPV**	** **	** **	
Kato-Katz	93.9% (89.0%, 98.3%)	92.1% (79.4%, 98.9%)	1.8% (-7.1%, 14.7%)
CCAtn	86.2% (80.5%, 91.3%)	91.1% (78.4%, 99.0%)	-5.0% (-15.1%, 8.6%)
CCAtp	64.3% (58.9%, 70.0%)	57.2% (45.8%, 69.5%)	7.1% (-6.1%, 19.8%)
CCAtn - Kato-Katz	-7.7% (-14.1%, -1.0%)	-1.0% (-14.4%, 11.7%)	
CCAtp - Kato-Katz	-29.6% (-36.2%, -22.6%)	-34.9% (-48.5%, -19.2%)	
CCAtp - CCAtn	-21.8% (-26.5%, -17.3%)	-33.9% (-45.1%, -21.9%)	
**NPV**	** **	** **	
Kato-Katz	90.5% (88.1%, 92.6%)	85.8% (79.8%, 90.6%)	4.6% (-0.8%, 11.0%)
CCAtn	88.2% (85.7%, 90.3%)	89.1% (82.9%, 93.8%)	-0.9% (-6.3%, 5.5%)
CCAtp	90.5% (88.2%, 92.4%)	92.2% (86.7%, 96.1%)	-1.7% (-6.4%, 4.0%)
CCAtn - Kato-Katz	-2.3% (-3.7%, -0.9%)	3.2% (0.9%, 5.5%)	
CCAtp - Kato-Katz	0.0% (-1.5%, 1.5%)	6.4% (3.3%, 9.6%)	
CCAtp - CCAtn	2.3% (1.5%, 3.2%)	3.1% (1.0%, 6.0%)	
**Test prevalence**		** **	
Kato-Katz	13.4% (12.4%, 14.4%)	6.1% (4.5%, 7.6%)	7.3% (5.4%, 9.1%)
CCAtn	11.7% (10.7%, 12.7%)	9.7% (7.8%, 11.5%)	2% (-0.1%, 4.1%)
CCAtp	20.1% (18.8%, 21.4%)	22.5% (19.8%, 25.3%)	-2.3% (-5.4%, 0.7%)
**Comparison of prevalence estimates**		** **	
Kato-Katz - infection prev	-7.2% (-9.7%, -4.7%)	-13.3% (-19.1%, -8.5%)	6.1% (0.6%, 12.5%)
CCAtn - infection prev	-8.8% (-11.4%, -6.5%)	-9.7% (-15.6%, -4.7%)	0.9% (-4.8%, 7.3%)
CCAtp - infection prev	-0.4% (-3.2%, 2.1%)	3.1% (-3.3%, 8.4%)	-3.5% (-9.5%, 3.2%)
CCAtn - Kato-Katz	-1.7% (-3.1%, -0.3%)	3.6% (1.2%, 6.1%)	-5.3% (-8.1%, -2.5%)
CCAtp - Kato-Katz	6.8% (5.2%, 8.3%)	16.4% (13.3%, 19.6%)	-9.6% (-13.2%, -6.1%)
CCAtp - CCAtn	8.4% (6.8%, 10.1%)	12.8% (9.4%, 16.2%)	-4.4% (-8.1%, -0.6%)

Sensitivity of Kato-Katz in Côte d’Ivoire (59.9%) was significantly higher than in Uganda (32.3%; Table 1, Fig 2), although, there was no evidence of sensitivity estimates of CCAtn and CCAtp differing between the countries (CCAtn = 49.3% and 50.1%; CCAtp = 63.0% and 69.7% in Côte d’Ivoire and Uganda respectively). In both countries, the sensitivity of CCAtp was significantly higher than the sensitivity of CCAtn. However, the countries differed with respect to the patterns of differences between CCA and Kato-Katz. In Côte d’Ivoire, the sensitivity of CCAtn was significantly lower than the sensitivity of Kato-Katz, but the sensitivity of CCAtp was not significantly different from the sensitivity of Kato-Katz. In contrast, in Uganda, the sensitivities of CCAtn and CCAtp were both higher than the sensitivity of Kato-Katz.

The estimated specificity of Kato-Katz did not differ significantly across the two countries (99.0% in Côte d’Ivoire vs. 99.3% in Uganda; Table 1, Fig 2). There was no evidence that the specificity of CCAtn differed between the countries (Côte d’Ivoire = 98.0%, Uganda = 98.8%), but the estimated specificity of CCAtp in Côte d’Ivoire (91.0%) was marginally, non-significantly, higher than in Uganda (87.6%; Table 2, Fig 2). In both countries, the estimated specificity of CCAtp was significantly less than both the estimated specificity of CCAtn and the estimated specificity of Kato-Katz. In Côte d’Ivoire, the estimated specificity of CCAtn was marginally, non-significantly, less than the estimated specificity of Kato-Katz and there was no evidence in Uganda that the estimated specificity of CCAtn and Kato-Katz differed.

### Comparison between infection and test prevalence

**Fig 3:**
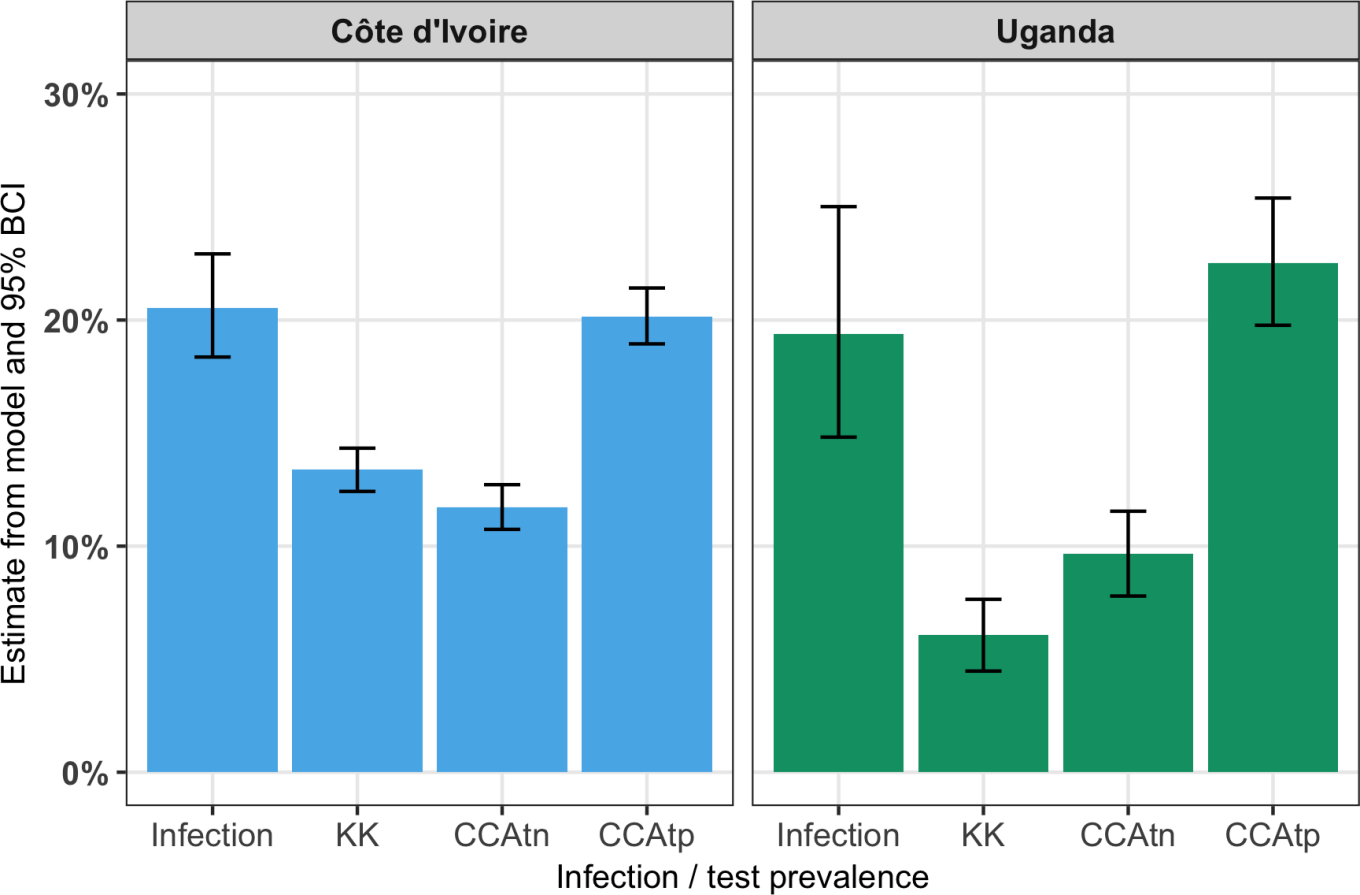
Estimated infection prevalence and tests prevalence from each test in Côte d’Ivoire and Uganda obtained from LCA.

Infection prevalence was estimated to be 20.5% in Côte d’Ivoire (Table 1; Fig 3) and 19.4% in Uganda. Indeed, there was no evidence that the estimates of infection prevalence differed between the countries (Table 2; Fig 3).

Both Kato-Katz and CCAtn substantially underestimated infection prevalence (Table 2; Fig 3). In contrast, there was no significant difference between test and infection prevalence by CCAtp in either country, with the CCAtp prevalence falling within 0.4 percentage points of the infection prevalence estimate in Côte d’Ivoire, and within 3.1 percentage points of the infection prevalence estimate in Uganda.

## Discussion

The aim of this study was to understand the implications considering CCA trace as negative or positive using data from two countries, Côte d’Ivoire and Uganda. We particularly focused on assessing the performance of CCA and Kato-Katz at measuring infection prevalence, as this is the main purpose of *S*. *mansoni* diagnostics in the control programs. We found that the sensitivities and specificities of CCA were much more consistent between countries than Kato-Katz and that estimates of prevalence by CCA with trace as positive was not significantly different from infection prevalence in either country. We discuss below possible reasons and implications for these results.

We found that trace values treated as positive (CCAtp) had significantly and substantially higher sensitivity and lower specificity than when treated as negative (CCAtn) in both countries. However, the sensitivity estimates indicated that CCAtp still did not detect a substantial proportion of infections (at least 30% in both countries), and this was supported by the raw data where 35% and 24% of the children that were Kato-Katz positive in Côte d’Ivoire and Uganda, respectively were negative by CCA. Although some of this may be due to misidentifying of samples, it seems extraordinary that this could explain the entire pattern.

We used LCA to analyse the data, as there is no ‘gold standard’ test for *S*. *mansoni* infection. We put a strong prior distribution (95% certain greater than 80%, with mode at 95%) on the specificity of Kato-Katz which may partly explain why estimated specificity of Kato-Katz (99%) was high and did not differ between countries. However, the lower Bayesian 95% confidence limit of Kato-Katz specificity was greater than the mode of the prior distribution suggesting that the data were not indicating lower specificity of the Kato-Katz than the prior. Consequently, the use of Bayesian analysis enabled us to assess the appropriateness of our assumptions, which would not be possible in a frequentist framework.

We elected to analyse trace positive and trace negative results within a single model. This method of analysis can be applied to any diagnostic where the result is not binary, where the results are in some way graded, and where alternative cut-off points can be assumed positive. The analysis is simple to implement and extend, with the key being that increasing the number of people testing positive on a sliding scale increases the sensitivity but decreases the specificity of the test. The preferred sensitivity and specificity balance of a diagnostic will depend on a number of different factors such as the properties of other diagnostics in use, the expected prevalence in the test population, and cost considerations. An alternative way to approach the analysis would be to analyse CCA trace negative and trace positive results separately. However, this risks the model returning logically impossible sensitivity estimates lower for trace positive than trace negative, or specificity estimates higher for trace positive than trace negative. Fitting trace positive and negative within a single model prevents this and also avoids having to interpret multiple estimates for sensitivities and specificities of other tests, and also for infection prevalence.

We analysed the data in a Bayesian framework, where the use of prior distributions can help overcome issues around degrees of freedom while still letting the data indicate if the assumptions made are not valid, as opposed to the absolute assumptions that can be required in a frequentist framework. Bayesian analysis also outputs the full distribution of each parameter through the iterations saved by the model. Consequently, distributions of additional variables calculated from the parameters are simple to obtain; we used this technique both to test for significance of differences between terms and between studies and to compare test prevalence and infection prevalence for each test.

Comparing between studies, we found that the estimated sensitivity and specificity of both CCAtp and CCAtn did not differ between the countries, in line with previous results that found the performance of CCA to be consistent before and after treatment [13]. However, the estimated sensitivity of Kato-Katz was much higher in Côte d’Ivoire, where the mean infection intensity was also much higher, than in Uganda. Lower sensitivity of Kato-Katz at low infection intensities is a well-known issue [10] and it is possible that this is the reason for our findings. A number of previous papers have assumed Kato-Katz to be the gold standard, including in a recent Cochrane review [27]. It is clear from these, and many other results, that it is not appropriate for Kato-Katz to be considered a gold standard, particularly at low intensities. We echo researchers in this [28] and other [29] fields in concluding that LCA is the most appropriate tool for assessing test sensitivity and specificity in the absence of a gold standard.

Our work adds to a growing body of literature using LCA to assess the performance of CCA [20, 30–35]. Our estimates of sensitivity for both CCA and Kato-Katz were on the lower end of those observed in these other studies, and our specificity estimates for CCA were relatively high. These studies together seem to be reflecting the general pattern of Kato-Katz sensitivity being strongly associated with infection intensities, with CCA perhaps being more consistent between environments [36]. However, this study is the first, to our knowledge, to assess the performance of CCA with respect to its main use in control programs of estimating prevalence within the study population.

Comparison of test and estimated infection prevalence suggested that the prevalence measured by CCAtp was not significantly different from infection prevalence in either country and that both Kato-Katz and CCAtn significantly underestimated infection prevalence in both countries. Consequently, our results imply that CCAtp is revealing substantial numbers of children infected with *S*. *mansoni* that were not detectable by Kato-Katz [37]. Although it could be argued that the infected children that are being missed by Kato-Katz are those with the lowest infection intensities, even low levels of schistosomiasis have an associated morbidity burden [38]. Interestingly, estimated infection prevalence did not differ between Côte d’Ivoire and Uganda, despite Kato-Katz prevalence being more than twice as high in Côte d’Ivoire than in Uganda. The difference in Kato-Katz prevalence was presumably due to repeated rounds of treatment in Uganda leading to lower average infection intensities than in Côte d’Ivoire, which are better detected by CCA than Kato-Katz. However, in the absence of historical Kato-Katz and CCA data from the schools in Uganda, it is not possible to assess whether repeated rounds of treatment in Uganda has also been associated with a concurrent decrease in estimated prevalence.

The main weakness of this study is the reliance on only two tests. Additional tests would be expected to increase the robustness of the study through increasing degrees of freedom, and there is clearly a need for additional studies incorporating more tests. However, we tried to mitigate for this weakness by using LCA, and also by incorporating covariances between the tests, which is expected to lead to more robust estimates than simply assuming the properties of different tests to be independent [39]. Additionally, the sample sizes in Côte d’Ivoire were over 4-fold higher than in Uganda, which is likely reflected in the larger confidence intervals around estimates from Uganda. As CCA becomes a more commonly used field tool, we expect sample sizes available for analyses to also increase.

We demonstrated here how ambiguous trace results can be incorporated into LCA using *S*. *mansoni* data from Côte d’Ivoire and Uganda, enabling direct comparison of test properties when trace was considered both as negative and positive, and avoiding having to make assumptions as to the nature of trace results. Our results suggested CCA with trace as positive was most reflective of infection prevalence and that both Kato-Katz and CCA with trace as negative substantially underestimated infection prevalence. Consequently, we conclude that CCA is an appropriate tool for field testing for *S*. *mansoni* in control programmes.

## Supporting information

S1 Supporting Information Detailed methodology of Bayesian latent class analysis. (DOCX)

S2 Supporting Information R2OpenBUGS code used to run the model. (DOCX)

S3 Supporting Information STARD checklist. (DOCX)

S4 Supporting Information Participant flow diagram. (PDF)

S5 Supporting Information Cote d’Ivoire data. (CSV)

S6 Supporting Information Uganda data. (CSV)

S7 Supporting Information Infection category cross tab. CCA results by WHO Kato-Katz infection category in each country.
(DOCX)
