# Supplementary material for: Interpreting ambiguous ‘trace’ results in Schistosoma mansoni CCA Tests: Estimating sensitivity and specificity of ambiguous results with no gold standard
Source: PLoS Negl Trop Dis. 2017 Dec 8;11(12):e0006102. doi: 10.1371/journal.pntd.0006102 (PMC5738141; doi:10.1371/journal.pntd.0006102)
Supplement: S1 Supporting Information — (DOCX) [file pntd.0006102.s001.docx]

**S1 Supporting Information: Detailed methodology of Bayesian Latent Class Analysis**

*Latent class analysis*

When there is no gold standard, latent class analysis can be used in order to estimates the sensitivity and specificity of each test, along with an estimate of infection prevalence in the population (reviewed in [26] and [21]). In the simplest form, with two tests, each being positive or negative, a two-by-two table is produced detailing the results found (S1 Supporting Information table 1.1).

|  | | **Test 2** | |
| --- | --- | --- | --- |
|  |  | **pos** | **neg** |
| **Test 1** | **pos** | $n_{++}$ | $n_{+-}$ |
|  | **neg** | $n_{-+}$ | $n_{--}$ |

S1 Supporting Information table 1.1

Here we use $n_{12}$ to denote the number of people with specific test combinations for test 1 and test 2 respectively. For example, $n_{++}$denotes the number of people testing positive for both test 1 and test 2, whereas $n_{+-}$ denotes the number of people testing positive for test 1 and negative for test 2. Note that there is no reference to whether or not an individual is truly positive or negative.

Latent class analysis is a method for relating the true infection status (the latent or unobserved variable) to the observed test status. Each box is taken in turn and the proportion of people in each category is related to the sensitivity and specificity of the tests and the true prevalence of infection in the population. Assuming the results of tests 1 and 2 are independent, conditional on the true infection status, we can put this together to obtain the following equations:

$$p_{++} =prev*{Se}_{1}*{Se}_{2}+\left( 1-prev \right)*\left( 1-{Sp}_{1} \right)*\left( 1-{Sp}_{2} \right) (1)$$

$$p_{+-} =prev*{Se}_{1}*{(1- Se}_{2})+\left( 1-prev \right)*\left( 1-{Sp}_{1} \right)*{Sp}_{2} (2)$$

$$p_{-+} =prev*{(1- Se}_{1})*{Se}_{2}+\left( 1-prev \right)*{Sp}_{1}*(1-{Sp}_{2}) (3)$$

$$p_{--} =prev*( 1-{Se}_{1})*{(1- Se}_{2})+\left( 1-prev \right)*{Sp}_{1}*{Sp}_{2} (4)$$

where$p_{12}$ denotes the proportion of people with specific test combinations; ${Se}_{1}$ and ${Se}_{2}$ denote the sensitivity of test 1 and test 2 respectively; ${Sp}_{1}$ and $;{Sp}_{2}$ denote the specificity of test 1 and test 2 respectively; and $prev$ denotes the infection prevalence in the population.

In words, equation 1 is equivalent to:

“The proportion of people testing positive for both tests equals the proportion of people in the population are truly infected and correctly test positive on both tests plus the proportion of people in population that are truly uninfected and incorrectly test positive on both tests.”

The data are then linked to equations 1 -4 through a multinomial distribution where $N$ is the total number of people tested:

$$\left( n_{++}, n_{+-}, n_{-+}, n_{--} \right) \sim Multinomial\left( p_{++}, p_{+-}, p_{-+}, p_{--}, N \right) (5)$$

In the simplest model described above, with two tests, there are three degrees of freedom (the number of test combinations minus 1), and five parameters to be estimated – the sensitivity and specificity of each test and the infection prevalence. The lack of degrees of freedom can be overcome by including data from at least one additional test into the analysis.

Analysis of the model can be in either a frequentist of Bayesian framework. The lack of degrees of freedom is accounted for in the frequentist framework by fixing the sensitivity or specificity of one or more test at a certain level, generally one. If the sensitivity and specificity of one test are both fixed at one, then this is equivalent to assuming that the test is a ‘gold standard’. Alternatively, in a Bayesian framework, the use of strong prior distributions perform a similar role while still enabling the data to indicate if the diagnostic test parameters are not as assumed.

Hui and Walter 1980 [27] described an extension to the basic model where the population is split into sub-populations and multiple comparison tables created, each having a total of three degrees of freedom. The sensitivity and specificity of each test is assumed constant across all populations, with differing infection prevalence in each population, and the number of parameters that have to be estimated is then equal to number of populations plus four. If a sufficient number of populations are included in the analysis, then there can be enough degrees of freedom to enable the parameters to be estimated.

*Non-independence between tests*

The models described above assume that the tests are conditionally independent – that is, conditioning upon the infection status, there is no relationship between the sensitivities or specificities of the tests. However, this assumption can easily be violated – for example when both tests are less likely to detect low intensity infections than high intensity infections. Dendukunari and Joseph (2001) proposed an extension to the model where a covariance term is included between the tests. For the example of two tests, the model would become:

$$p_{++} =prev*{(Se}_{1}*{Se}_{2}+ covSe)+\left( 1-prev \right)*(\left( 1-{Sp}_{1} \right)*\left( 1-{Sp}_{2} \right)+ covSp) (6)$$

$$p_{+-} =prev*{(Se}_{1}*{(1- Se}_{2})- covSe)+\left( 1-prev \right)*(\left( 1-{Sp}_{1} \right)*{Sp}_{2}-covSp) (7)$$

$$p_{-+} =prev*{((1- Se}_{1})*{Se}_{2}-covSe)+\left( 1-prev \right)*{(Sp}_{1}*(1-{Sp}_{2})-covSp) (8)$$

$$p_{--} =prev*\left( \left( 1-{Se}_{1} \right)*{(1- Se}_{2} \right)+ coveSe)+\left( 1-prev \right)*{(Sp}_{1}*{Sp}_{2}+covSp) (9)$$

where $covSe$ denotes the covariance in sensitivities between the two tests; $covSp$ denotes the covariance in specificities between the two tests; and all other parameters remain the same as described previously. In this model if the tests are positively correlated, the proportion of people testing the same in both tests (++ or --) is inflated by the covariance amount while the proportion of people testing differently in each test (+- or -+) is deflated by the covariance amount.

The addition of the covariance term requires that limits be placed on the covariance so that the term multiplied by the prevalence (or 1 minus the prevalence) remains bounded between 0 and 1. The limits are entered into the model by specifying a uniform prior distribution on the range:

$$\left( {Se}_{1}-1 \right)(1- {Se}_{2}) \leq covSe \leq\min\left( {Se}_{1}, {Se}_{2} \right)-{Se}_{1}*{Se}_{2} (10)$$

$$\left( {Sp}_{1}-1 \right)(1- {Sp}_{2}) \leq covSp \leq\min\left( {Sp}_{1}, {Sp}_{2} \right)-{Sp}_{1}*{Sp}_{2} (11)$$

*Extension of the model to ambiguous test results*

Here we extend the model of Dendukuri and Joseph to instances when there is uncertainty in the test result, for example, when there is a trace result that can be considered as either negative or positive, or different degrees of positive results. In this instance, we add one or more columns or rows for the tests with more than two possible test results. S1 Supporting Information table 1.2 shows S1 Supporting Information table 1.1 extended to include a trace result for test 2.

|  | | **Test 2** | | |
| --- | --- | --- | --- | --- |
|  |  | **pos** | **tr** | **neg** |
| **Test 1** | **pos** | $n_{++}$ | $n_{+t}$ | $n_{+-}$ |
|  | **neg** | $n_{-+}$ | $n_{-t}$ | $n_{--}$ |

S1 Supporting Information table 1.2

To incorporate this extra column, we consider the properties of the tests. In the example that we apply the method to, a CCA trace result can be considered as positive or negative. Consequently, the difference in the analysis lies in the treatment of the trace results. Assuming CCA trace to be positive (CCAtp) will lead to more people being classified positive than assuming trace to be negative (CCAtn). If any of these trace-positive patients are truly positive, then the CCAtp will be more sensitive than CCAtn. However, if any of these trace-positive patients are truly negative, then the CCAtp will be less specific than CCAtn. Therefore, we can say that:

- The sensitivity of the test with trace assumed to be positive must be *equal to or greater than* the sensitivity of the test with trace assumed to be negative.
- The specificity of the test with trace assumed to be positive must be *equal to or less than* the specificity of the test with trace assumed to be negative.

Equations 5-8 can then be extended to:

$$p_{++} =prev*{(Se}_{1}*{Se}_{2}+ {covSe}_{1, 2})+\left( 1-prev \right)*(\left( 1-{Sp}_{1} \right)*\left( 1-{Sp}_{2} \right)+ {covSp}_{1, 2}) (12)$$

$$p_{+t} =prev*{(Se}_{1}*adj{Se}_{2}{+ covSe}_{1,adj})+\left( 1-prev \right)*(\left( 1-{Sp}_{1} \right)*adj{Sp}_{2}{+ covSp}_{1,adj}) (13)$$

$$p_{+-} =prev*{(Se}_{1}*{(1-(Se}_{2}+ adj{Se}_{2})-({covSe}_{1, 2}+ {covSe}_{1,adj}))+\left( 1-prev \right)*(\left( 1-{Sp}_{1} \right)*{(Sp}_{2}- adj{Sp}_{2}) -({covSp}_{1, 2}- {covSp}_{1,adj})) (14)$$

$$p_{-+} =prev*{((1- Se}_{1})*{Se}_{2}-{covSe}_{1, 2})+\left( 1-prev \right)*{(Sp}_{1}*(1-{Sp}_{2})-{covSp}_{1, 2}) (15)$$

$$p_{-t} =prev*{((1- Se}_{1})*adj{Se}_{2}-{covSe}_{1,adj})+\left( 1-prev \right)*{(Sp}_{1}*adj{Sp}_{2}{- covSp}_{1,adj}) (16)$$

$p_{--} =prev*\left( \left( 1-{Se}_{1} \right)*{(1-(Se}_{2}+ adj{Se}_{2} \right))+ ({covSe}_{1, 2}+ {covSe}_{1,adj}))+\left( 1-prev \right)*{(Sp}_{1}*{(Sp}_{2}- adj{Sp}_{2})+ ({covSp}_{1, 2}- {covSp}_{1,adj})) (17)$

where ${Se}_{2}$ and ${Sp}_{2}$is the specificity and specificity of CCAtn respectively. The sensitivity of CCAtp is then equal to ${Se}_{2}+ adj{Se}_{2}$, and the specificity of CCAtp equals${Sp}_{2}- adj{Sp}_{2}$.

In a similar manner to Dendukuri and Joseph, we place limits on the covariances:

$${\left( {Se}_{1}-1 \right)(1- {Se}_{2}) \leq covSe}_{1, 2} \leq\min\left( {Se}_{1},{Se}_{2} \right)-{Se}_{1}*{Se}_{2} (18)$$

$${\left( {Se}_{1}-1 \right)(1- {Se}_{adj}) \leq covSe}_{1,adj} \leq\min\left( {Se}_{1},{adjSe}_{2} \right)-{Se}_{1}*{adjSe}_{2} (19)$$

$${{-Se}_{1}*({{Se}_{2}+adjSe}_{2}) \leq{covSe}_{1, 2}+ covSe}_{1,adj} \leq\min\left( {1-Se}_{1},{1-({Se}_{2}+adjSe}_{2} \right))-{(1-Se}_{1})*\left( {1-({Se}_{2}+adjSe}_{2} \right) (20)$$

$${\left( {Sp}_{1}-1 \right)(1- {Sp}_{2}) \leq covSp}_{1, 2} \leq\min\left( {Sp}_{1},{Sp}_{2} \right)-{Sp}_{1}*{Sp}_{2}(21)$$

$${\left( {Sp}_{1}-1 \right)(1- adj{Sp}_{2}) \leq covSp}_{1,adj} \leq\min\left( {Sp}_{1},{adjSp}_{2} \right)-{Sp}_{1}*adj{Sp}_{2}(22)$$

$${\left( {Sp}_{1}-1 \right)*\left( {1-({Sp}_{2}-adjSp}_{2} \right))\leq{covSp}_{1, 2}- covSp}_{1,adj} \leq\min\left( {Sp}_{1},{{Sp}_{2}-adjSp}_{2} \right)-{Sp}_{1}*{{(Sp}_{2}-adjSp}_{2}) (23)$$

and we also place limits on ${adjSe}_{2}$and ${adjSp}_{2}$so that the sensitivity and specificity of ${Se}_{2}+{adjSe}_{2}$and ${Sp}_{2}-{adjSp}_{2}$remain between 0 and 1:

$$0{\leq adjSe}_{2}\leq1- {Se}_{2} (24)$$

$$0{\leq adjSp}_{2}\leq{Sp}_{2} (25)$$

We incorporated the limits on the sum of the covariances into the model by adding extra restrictions to the individual covariances so that their sum was limited to the required amount.
