# Supplementary material for: Interpreting ambiguous ‘trace’ results in Schistosoma mansoni CCA Tests: Estimating sensitivity and specificity of ambiguous results with no gold standard
Source: PLoS Negl Trop Dis. 2017 Dec 8;11(12):e0006102. doi: 10.1371/journal.pntd.0006102 (PMC5738141; doi:10.1371/journal.pntd.0006102)
Supplement: S2 Supporting Information — (DOCX) [file pntd.0006102.s002.docx]

**S2 Supporting Information: R2OpenBUGS code used to run the model**

uganda.model <- function(){

#====likelihood==========================

for (i in 1:14){

y[i, 1] <- prev[i] * (Se.K * Se.C + cov.Se.K.C) + (1 - prev[i]) * ((1 - Sp.K) * (1 - Sp.C) + cov.Sp.K.C) # CCA determined by trace negative

y[i, 2] <- prev[i] * (Se.K * adjSe + cov.Se.K.adj) + (1 - prev[i]) * ((1 - Sp.K) * adjSp + cov.Se.K.adj) # CCA determined by difference between trace neg and pos

y[i, 3] <- prev[i] * (Se.K * (1 - (Se.C + adjSe)) - (cov.Se.K.C + cov.Se.K.adj)) + (1 - prev[i]) * ((1 - Sp.K) * (Sp.C - adjSp) - (cov.Sp.K.C - cov.Sp.K.adj)) # CCA determined by trace positive

y[i, 4] <- prev[i] * ((1 - Se.K) * Se.C - cov.Se.K.C) + (1 - prev[i]) * (Sp.K * (1 - Sp.C) - cov.Sp.K.C) # CCA determined by trace negative

y[i, 5] <- prev[i] * ((1 - Se.K) * adjSe - cov.Se.K.adj) + (1 - prev[i]) * (Sp.K * adjSp - cov.Sp.K.adj) # CCA determined by difference between trace neg and pos

y[i, 6] <- prev[i] * ((1 - Se.K) * (1 - (Se.C + adjSe)) + (cov.Se.K.C + cov.Se.K.adj)) + (1 - prev[i]) * (Sp.K * (Sp.C - adjSp) + (cov.Sp.K.C - cov.Sp.K.adj)) # CCA determined by trace positive

# tying y to the data

result[i, 1 : 6] ~ dmulti(y[i, 1 : 6], tested[i]) # observed data

}

#===prior distributions ===============================================

for (i in 1 : 14 ) {prev[i] ~ dbeta(1, 1) }

Se.K ~ dbeta(3.05, 1.51) # p2

Sp.K ~ dbeta(21.20, 2.06) # p6

Se.C ~ dbeta(3.05, 1.51) # p2

Sp.C ~ dbeta(5.38, 1.49) # p4

adjSe ~ dunif(0, U.adjSe)

adjSp ~ dunif(0, U.adjSp)

cov.Se.K.C ~ dunif(0, U.cov.Se.K.C)

cov.Se.K.adj ~ dunif(0, U.cov.Se.K.adj)

cov.Sp.K.C ~ dunif(0, U.cov.Sp.K.C)

cov.Sp.K.adj ~ dunif(0, U.cov.Sp.K.adj)

# limits for the priors

U.adjSe <- 1 - Se.C

U.adjSp <- Sp.C

# sensitivity covariances

U.cov.Se.K.C <- min(

min(Se.K, Se.C) - Se.K * Se.C,

min((1 - Se.K), (1 - (Se.C + adjSe))) - (1 - Se.K) * (1 - (Se.C + adjSe)))

U.cov.Se.K.adj <- min(

min(Se.K, adjSe) - Se.K * adjSe,

min((1 - Se.K), (1 - (Se.C + adjSe))) - (1 - Se.K) * (1 - (Se.C + adjSe)) - cov.Se.K.C)

U.covKtpSe <- min(Se.K, (Se.C + adjSe)) - Se.K * (Se.C + adjSe)

# specificity covariances

U.cov.Sp.K.C <- min(

min(Sp.K, Sp.C) - Sp.K * Sp.C,

min(Sp.K, (Sp.C - adjSp)) - Sp.K * (Sp.C - adjSp) + cov.Sp.K.adj

)

U.cov.Sp.K.adj <- min(Sp.K, adjSp) - Sp.K * adjSp

U.covKtpSp <- min(Sp.K, (Sp.C - adjSp)) - Sp.K * (Sp.C - adjSp)

}

uganda.inits <- function(){list(prev = runif(14, 0, 1),

Se.K = runif(1, 0.2, 0.8),

Sp.K = runif(1, 0.9, 1),

Se.C = runif(1, 0.4, 1),

Sp.C = runif(1, 0.6, 1),

adjSe = runif(1, 0, 0.19), # have to make sure that Se.C + adjSe < 1

adjSp = runif(1, 0.01, 0.4), # have to make sure that Se.C - adjSe > 0

cov.Se.K.C = 0,

cov.Se.K.adj = 0,

cov.Sp.K.C = 0,

cov.Sp.K.adj = 0

)}

uganda.monitor <- c("prev",

"Se.K", "Sp.K",

"Se.C", "Sp.C",

"adjSe", "adjSp",

"cov.Se.K.C", "cov.Sp.K.C",

"cov.Se.K.adj", "cov.Sp.K.adj",

"Se.Cp", "Sp.Cp",

"cov.Se.K.Cp", "cov.Sp.K.Cp",

)

uganda.out <- bugs(uganda.data, uganda.inits, uganda.monitor, uganda.model,

n.iter=3000, n.burnin=1000, n.thin=25, n.chains=3, debug = T)
