# Supplementary material for: Interpreting ambiguous ‘trace’ results in Schistosoma mansoni CCA Tests: Estimating sensitivity and specificity of ambiguous results with no gold standard
Source: PLoS Negl Trop Dis. 2017 Dec 8;11(12):e0006102. doi: 10.1371/journal.pntd.0006102 (PMC5738141; doi:10.1371/journal.pntd.0006102)
Supplement: S3 Supporting Information — (DOCX) [file pntd.0006102.s003.docx]

|  | **Section & Topic** | **No** | **Item** | **Details** |
| --- | --- | --- | --- | --- |
|  |  |  |  |  |
|  | **TITLE OR ABSTRACT** |  |  |  |
|  |  | **1** | Identification as a study of diagnostic accuracy using at least one measure of accuracy (such as sensitivity, specificity, predictive values, or AUC) | Manuscript title contains the words ‘sensitivity’ and ‘specificity’ |
|  | **ABSTRACT** |  |  |  |
|  |  | **2** | Structured summary of study design, methods, results, and conclusions  (for specific guidance, see STARD for Abstracts) | Abstract structured as background, methodology/principle findings, conclusions |
|  | **INTRODUCTION** |  |  |  |
|  |  | **3** | Scientific and clinical background, including the intended use and clinical role of the index test | See introduction |
|  |  | **4** | Study objectives and hypotheses | “The aim of this study is to robustly analyse CCA data from two countries, Côte d’Ivoire and Uganda, to determine the effects of considering CCA trace as negative or positive. We particularly focus on assessing the performance of CCA and Kato-Katz at measuring ‘infection prevalence’, which is estimated from the LCA, and is the main use of S. mansoni diagnostics in control programs.” |
|  | **METHODS** |  |  |  |
|  | *Study design* | **5** | Whether data collection was planned before the index test and reference standard  were performed (prospective study) or after (retrospective study) | “In Côte d’Ivoire…the sample size calculation was powered to be able to detect change in prevalence between the baseline and repeat surveys and not for the purposes of diagnostic comparison. In Uganda… 14 schools were sampled using both CCA and Kato-Kato to obtain a pre-determined sample size of approximately 300 pupils to enable comparison of test results with LCA” |
|  | *Participants* | **6** | Eligibility criteria | “In Côte d’Ivoire the sampling frame was all schools involved in a baseline schistosomiasis mapping survey in 2012 where at least one child infected with S. mansoni was observed during mapping, within districts that fell into moderate (≥ 10% and <50% prevalence by Kato-Katz) or high (≥ 50% prevalence by Kato-Katz) prevalence categories by WHO guidelines [19]. Mapped schools were used in the site selection as no treatment had occurred in these schools post-mapping.  In Uganda, 120 schools were selected to be were sampled using CCA only, with 40 schools being in each of three treatments arms, although in the field only 119 schools were sampled. A subset of 14 schools (planned to be 15) were sampled using both CCA and Kato-Katz to obtain a pre-determined sample size of approximately 300 pupils in each treatment arm to enable comparison of test results using LCA; here we focus on the 14 schools only. The sampling frame for Uganda was a list of all mixed-sex primary schools with at least 100 pupils in ‘low endemic’ sub districts from the 2012 CCA mapping exercise in Uganda, that had received at least four rounds of praziquantel treatment. Schools that were mapped as part of the recent mapping exercise where no schistosomiasis was found with CCA were excluded from the sampling frame and ‘low endemic’ was defined as those subcounties with equivalent to less than 10% S. mansoni prevalence by Kato-Katz, back calculated as 1-46% by CCA [20]. ” |
|  |  | **7** | On what basis potentially eligible participants were identified  (such as symptoms, results from previous tests, inclusion in registry) | “In Côte d’Ivoire, 125 children per school from classes CP1, CP2 and CE1 (roughly equivalent to 6, 7 and 8-year-olds) were sampled. In Uganda, 60 students were sampled in each school although sampling of children by grades was not consistent. Nine schools sampled children in primary classes 1, 4, 5 and 6 (roughly equivalent to 6, 9, 10 and 11-year-olds) as per the sampling protocol and five schools deviated from the sampling protocol by sampling children in grades 1, 3, 5 and 7 (roughly equivalent to 6, 8, 10 and 12-year-olds). In both countries, boys and girls were randomly sampled within each school, selection of pupils within each class and sex was random, and there was no reference to possible infection status during selection” |
|  |  | **8** | Where and when potentially eligible participants were identified (setting, location and dates) | “Data were collected as part of the programme Monitoring and Evaluation activities within each country. Samples were collected as part of a Monitoring & Evaluation impact study, in which a consistent age range of children in a set of schools (sentinel sites) are screened for schistosomiasis over multiple years to estimate changes in prevalence and intensity of infection over time. In Côte d’Ivoire data collected in 2013 were ‘baseline’ data, that is, data collected in the first year of the control programme prior to mass treatment with Praziquantel. However, Uganda established a national schistosomiasis control programme in 2003 and had received at least four rounds of PC prior to the survey in 2013. In addition, schools selected in Uganda were part of an operational research project where three different treatment strategies in years following the baseline survey were randomly allocated to each of the schools. Data were collected between 28th October – 12th November 2013 in Côte d’Ivoire and between 30th September – 16th October 2013 in Uganda.” |
|  |  | **9** | Whether participants formed a consecutive, random or convenience series | “The surveyed schools were randomly selected from the sampling frame in each country. In both countries, equal number of boys and girls were randomly sampled within each school, selection of pupils within each class and sex was random, and there was no reference to possible infection status during selection.” |
|  | *Test methods* | **10a** | Index test, in sufficient detail to allow replication | “CCA data were collected on the first day of the survey in Côte d’Ivoire and on all three days in Uganda; for comparison purposes only CCA results from the first day of data collection have been assessed in both countries. CCA results were scored on a four point scale in Côte d’Ivoire and a five point scale in Uganda, with 0 denoting negative and the lowest positive score denoting trace, as per manufacturers recommendation, in both countries. A child was defined to be positive by CCA with trace assumed negative (CCAtn) if the CCA result was greater than trace in either country, and positive by CCA with trace assumed positive (CCAtp) if the CCA result was greater than or equal to trace in either country.” |
|  |  | **10b** | Reference standard, in sufficient detail to allow replication | “Duplicate Kato-Katz thick smear slides per stool sample (using 41.7 mg template) were prepared and examined for S. mansoni using WHO approved standard operating procedures on each of two and three consecutive days in Côte d’Ivoire and Uganda, respectively. To provide a like-for-like comparison, we included data collected on only the first two days from Uganda in results presented here. A child was defined to be positive by Kato-Katz if one or more S. mansoni eggs were found on any of the four slides examined in total, and mean infection intensity in eggs per gram (epg) was calculated by multiplying the average number of eggs found across all examined slides by 24.” |
|  |  | **11** | Rationale for choosing the reference standard (if alternatives exist) | “Frequency of treatment and who receives the drugs are dependent on the prevalence of schistosomiasis in the local area [6], as determined by the parasitological diagnostic test Kato-Katz, where eggs are detected in faecal samples examined microscopically [7, 8].” |
|  |  | **12a** | Definition of and rationale for test positivity cut-offs or result categories  of the index test, distinguishing pre-specified from exploratory | “CCA results were scored on a four point scale in Côte d’Ivoire and a five point scale in Uganda, with 0 denoting negative and the lowest positive score denoting trace, as per manufacturers recommendation, in both countries. A child was defined to be positive by CCA with trace assumed negative (CCAtn) if the CCA result was greater than trace in either country, and positive by CCA with trace assumed positive (CCAtp) if the CCA result was greater than or equal to trace in either country.” |
|  |  | **12b** | Definition of and rationale for test positivity cut-offs or result categories  of the reference standard, distinguishing pre-specified from exploratory | “A child was defined to be positive by Kato-Katz if one or more *S. mansoni* eggs were found on any of the four slides examined” |
|  |  | **13a** | Whether clinical information and reference standard results were available  to the performers/readers of the index test | “Kato-Katz and CCA tests processing was performed by the same survey team in each school. The tests were numbered and the Kato-Katz and CCA tests were processed separately. Consequently, clinical information and other tests results were not available during test processing.” |
|  |  | **13b** | Whether clinical information and index test results were available  to the assessors of the reference standard | “Kato-Katz and CCA tests processing was performed by the same survey team in each school. The tests were numbered and the Kato-Katz and CCA tests were processed separately. Consequently, clinical information and other tests results were not available during test processing.” |
|  | *Analysis* | **14** | Methods for estimating or comparing measures of diagnostic accuracy | “Analysis of the test results was by Bayesian Latent Class Analysis.” |
|  |  | **15** | How indeterminate index test or reference standard results were handled | “Only children with four slides of Kato-Katz (two slides on each of two days) and one CCA result were included in the analyses..” |
|  |  | **16** | How missing data on the index test and reference standard were handled | “Only children with four slides of Kato-Katz (two slides on each of two days) and one CCA result were included in the analyses.” |
|  |  | **17** | Any analyses of variability in diagnostic accuracy, distinguishing pre-specified from exploratory | “Analysis of the test results was by Bayesian LCA. Full details of the methodology are available in the Supplementary Information 1 and consequently we focus on the main points here. All analyses were pre-specified.” |
|  |  | **18** | Intended sample size and how it was determined | “In Côte d’Ivoire, all schools in the baseline survey were tested with both CCA and Kato-Katz, and the sample size calculation was powered to be able to detect change in prevalence between the baseline and repeat surveys and not for the purposes of diagnostic comparison… In Uganda, 120 schools were selected to be were sampled using CCA only, with 40 schools being in each of three treatments arms, although in the field only 119 schools were sampled. A subset of 14 schools (planned to be 15) were sampled using both CCA and Kato-Katz to obtain a pre-determined sample size of approximately 300 pupils in each treatment arm to enable comparison of test results using LCA; here we focus on the 14 schools only.” |
|  | **RESULTS** |  |  |  |
|  | *Participants* | **19** | Flow of participants, using a diagram | See supplementary information 4 |
|  |  | **20** | Baseline demographic and clinical characteristics of participants | See Table 1 |
|  |  | **21a** | Distribution of severity of disease in those with the target condition | See supplementary information 7 |
|  |  | **21b** | Distribution of alternative diagnoses in those without the target condition | n/a as “In both countries, equal number of boys and girls were randomly sampled within each school, selection of pupils within each class and sex was random, and there was no reference to possible infection status during selection.“ |
|  |  | **22** | Time interval and any clinical interventions between index test and reference standard | “Duplicate Kato-Katz thick smear slides per stool sample (using 41.7 mg template) were prepared and examined for *S. mansoni* using WHO approved standard operating procedures on each of two and three consecutive days in Côte d’Ivoire and Uganda, respectively. To provide a like-for-like comparison, we included data collected on only the first two days from Uganda in results presented here…. CCA data were collected on the first day of the survey in Côte d’Ivoire and on all three days in Uganda; for comparison purposes only CCA results from the first day of data collection have been assessed in both countries.” |
|  | *Test results* | **23** | Cross tabulation of the index test results (or their distribution)  by the results of the reference standard | Table 1 |
|  |  | **24** | Estimates of diagnostic accuracy and their precision (such as 95% confidence intervals) | Table 2 |
|  |  | **25** | Any adverse events from performing the index test or the reference standard | “Testing required only urine and stool samples from each child and no adverse effects from performing either test were observed.” |
|  | **DISCUSSION** |  |  |  |
|  |  | **26** | Study limitations, including sources of potential bias, statistical uncertainty, and generalisability | “The main weakness of this study is the reliance on only two tests. Additional tests would be expected to increase the robustness of the study through increasing degrees of freedom, and there is clearly a need for additional studies incorporating more tests. However, we tried to mitigate for this weakness by using LCA, and also by incorporating covariances between the tests, which is expected to lead to more robust estimates than simply assuming the properties of different tests to be independent. Additionally, the sample sizes in Côte d’Ivoire were over 4-fold higher than in Uganda, which is likely reflected in the larger confidence intervals around estimates from Uganda. As CCA becomes a more commonly used field tool, we expect sample sizes for analyses to also increase. ” |
|  |  | **27** | Implications for practice, including the intended use and clinical role of the index test | “Our results suggested CCA with trace as positive was most reflective of infection prevalence and that both Kato-Katz and CCA with trace as negative substantially underestimated infection prevalence. Consequently, we conclude that CCA is an appropriate tool for field testing for S. mansoni in control programmes.” |
|  | **OTHER INFORMATION** |  |  |  |
|  |  | **28** | Registration number and name of registry | “Ethical approval for both surveys, including the consent process, was obtained from Imperial College Research Ethics Committee (ICREC_8_2_2) as well as from the appropriate country: Comité National d’Ethique de la Recherche (CNER; ref: 086/MSHP/CNER-kp) in Côte d’Ivoire and Uganda National Council for Science and Technology (UNCST; ref: HS1993) in Uganda.” |
|  |  | **29** | Where the full study protocol can be accessed | “Full study protocols are available on request from the Schistosomiasis Control Initiative, Imperial College London.” |
|  |  | **30** | Sources of funding and other support; role of funders | See funding statement as part of submission.  “This work was funded by the Department of International Development, UK (https://www.gov.uk/government/organisations/department-for-international-development), under the Integrated Control of Schistosomiasis and Intestinal Helminths in Sub-Saharan Africa (ICOSA) award PO 5309.” |
|  |  |  |  |  |
