# Supplementary figures and images for: Interpreting ambiguous ‘trace’ results in Schistosoma mansoni CCA Tests: Estimating sensitivity and specificity of ambiguous results with no gold standard
Source: PLoS Negl Trop Dis. 2017 Dec 8;11(12):e0006102. doi: 10.1371/journal.pntd.0006102 (PMC5738141; doi:10.1371/journal.pntd.0006102)

## Flow diagram for Cote d'Ivoire and Uganda

### Cote d'Ivoire

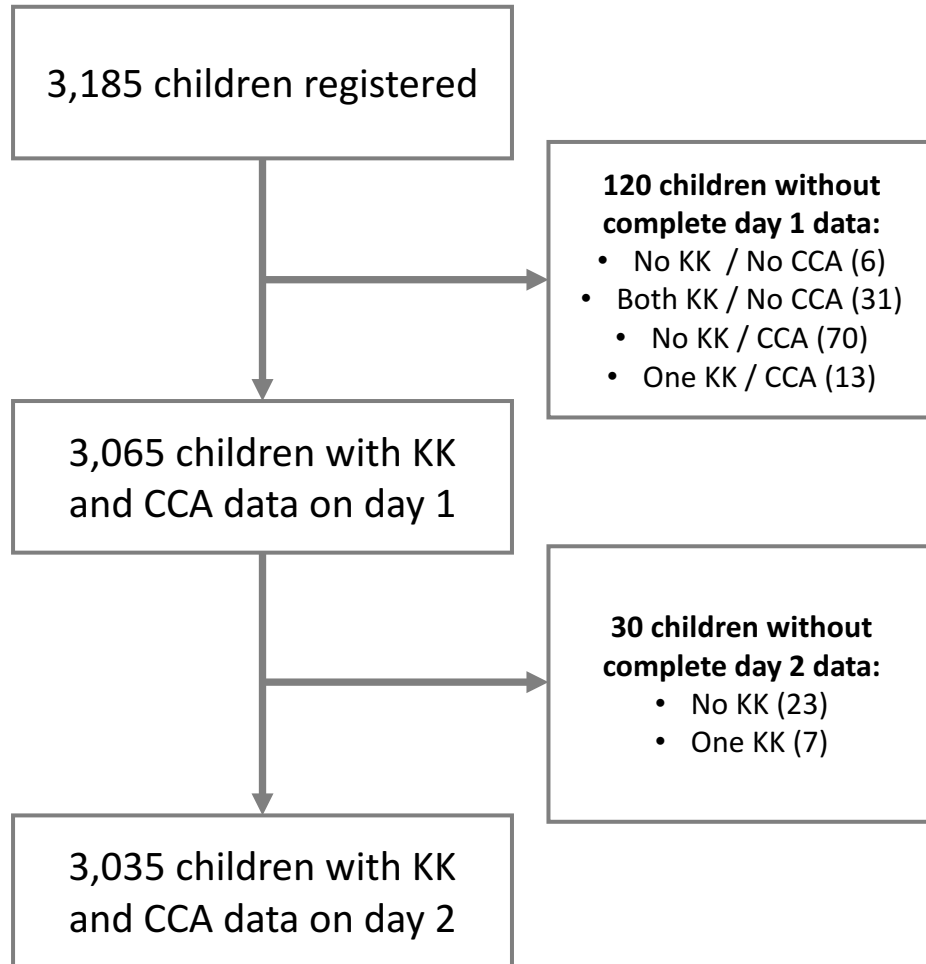

### Uganda

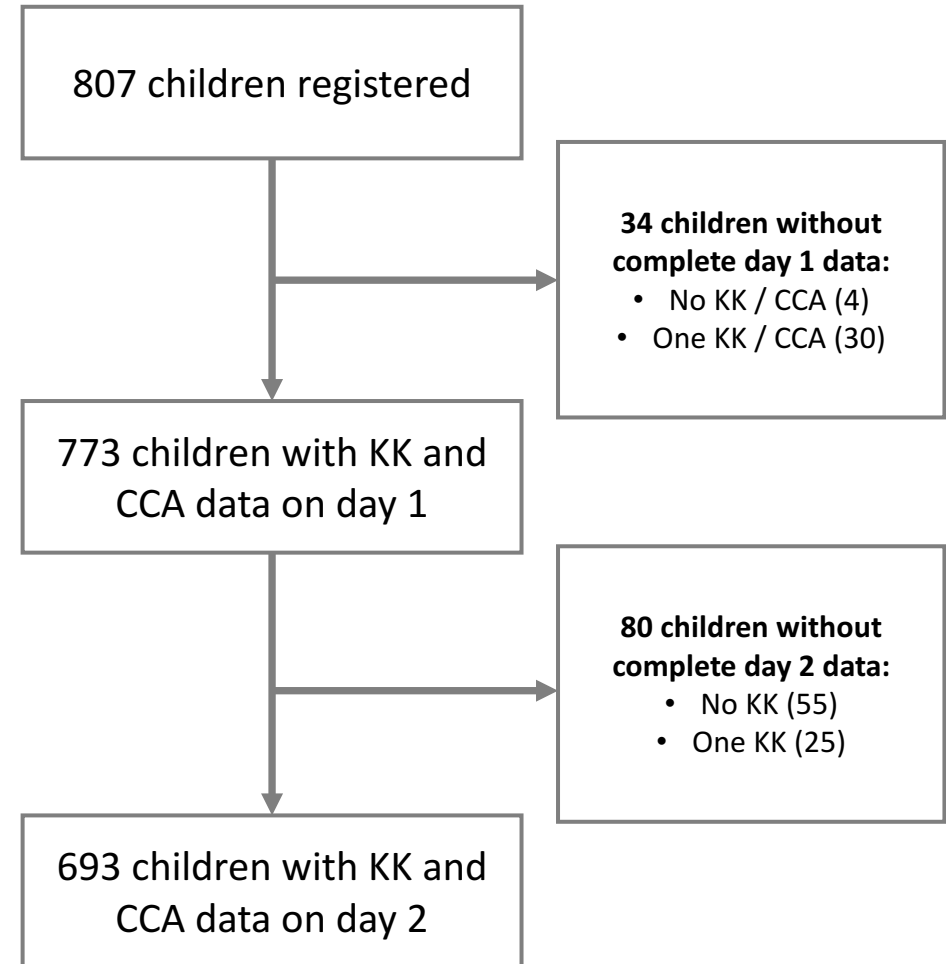

Supplement: S4 Supporting Information — (PDF) [file pntd.0006102.s004.pdf]
