# Supplementary material for: Interpreting ambiguous ‘trace’ results in Schistosoma mansoni CCA Tests: Estimating sensitivity and specificity of ambiguous results with no gold standard
Source: PLoS Negl Trop Dis. 2017 Dec 8;11(12):e0006102. doi: 10.1371/journal.pntd.0006102 (PMC5738141; doi:10.1371/journal.pntd.0006102)
Supplement: S7 Supporting Information — CCA results by WHO Kato-Katz infection category in each country. (DOCX) [file pntd.0006102.s007.docx]

| **Cote d'Ivoire** | | CCA result | | | |
| --- | --- | --- | --- | --- | --- |
|  |  | **0** | **tr** | **1** | **2** |
| Kato-Katz infection category by WHO guidelines | **uninfected** | 2282 | 188 | 124 | 35 |
|  | **light** | 115 | 55 | 67 | 22 |
|  | **moderate** | 23 | 13 | 35 | 28 |
|  | **heavy** | 4 | 0 | 23 | 21 |

| **Uganda** | | CCA result | | | | |
| --- | --- | --- | --- | --- | --- | --- |
|  |  | **0** | **tr** | **1** | **2** | **3** |
| Kato-Katz infection category by WHO guidelines | **uninfected** | 526 | 82 | 28 | 10 | 0 |
|  | **light** | 11 | 7 | 9 | 13 | 4 |
|  | **moderate** | 0 | 0 | 0 | 0 | 2 |
|  | **heavy** | 0 | 0 | 0 | 0 | 1 |
